# Senolytics alleviate the degenerative disorders of temporomandibular joint in old age

**DOI:** 10.1111/acel.13394

**Published:** 2021-06-08

**Authors:** Yueying Zhou, Iman M. A. Al‐Naggar, Po‐Jung Chen, Nathan S. Gasek, Ke Wang, Shivam Mehta, George A. Kuchel, Sumit Yadav, Ming Xu

**Affiliations:** ^1^ Xiangya Stomatological Hospital and School of Stomatology Central South University Changsha Hunan China; ^2^ UConn Center on Aging UConn Health Farmington CT USA; ^3^ Center for Regenerative Medicine and Skeletal Development UConn Health Farmington CT USA; ^4^ Division of Orthodontics UConn Health Farmington CT USA; ^5^ Department of Genetics and Genome Sciences UConn Health Farmington CT USA

**Keywords:** aging, cellular senescence, dasatinib, quercetin, TMJ

## Abstract

Aging is one of the major risk factors for degenerative joint disorders, including those involving the temporomandibular joint (TMJ). TMJ degeneration occurs primarily in the population over 65, significantly increasing the risk of joint discomfort, restricted joint mobility, and reduced quality of life. Unfortunately, there is currently no effective mechanism‐based treatment available in the clinic to alleviate TMJ degeneration with aging. We now demonstrate that intermittent administration of senolytics, drugs which can selectively clear senescent cells, preserved mandibular condylar cartilage thickness, improved subchondral bone volume and turnover, and reduced Osteoarthritis Research Society International (OARSI) histopathological score in both 23‐ to 24‐month‐old male and female mice. Senolytics had little effect on 4 months old young mice, indicating age‐specific benefits. Our study provides proof‐of‐concept evidence that age‐related TMJ degeneration can be alleviated by pharmaceutical intervention targeting cellular senescence. Since the senolytics used in this study have been proven relatively safe in recent human studies, our findings may help justify future clinical trials addressing TMJ degeneration in old age.

## Graphical abstract

Senolytic drugs preserved mandibular condylar cartilage thickness, improved subchondral bone volume and turnover, and reduced pathologies in old mouse TMJs.

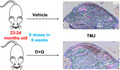

Aging is tightly associated with joint disorders of both knee and the temporomandibular joint (TMJ), which jointly contribute to musculoskeletal disability (Loeser, 2010), as well as decreased quality of life, pain, and potential nutritional deficits. The prevalence of TMJ degeneration is high in the older population, with a prevalence of 45%–70% in individuals over 65 (Schmitter et al., 2010). With aging, progressive loss of structure, function, coordination, and physiological integrity occurs in the mandibular condylar cartilage (MCC) and the subchondral bone of the TMJ, leading to TMJ degeneration(Chen et al., 2020). Degenerative disorders of the TMJ can cause severe pain, joint immobility, and compromised quality of life in patients (Trize et al., 2018), especially in older population with reduced resilience.

Compared with knee and other articular joints, TMJ has unique fibrocartilage with distinct developmental origin and different molecular composition, structure, and mineral content (Benjamin & Ralphs, 2004). The underlying cellular mechanism behind TMJ degeneration with aging is largely unknown. Moreover, no disease‐modifying drug is available to alleviate or prevent TMJ degeneration with aging. Emerging evidence indicates that targeting cellular senescence could delay the fundamental aging process and simultaneously alleviate a range of age‐related diseases (the Geroscience Hypothesis (Sierra & Kohanski, 2017)). Cellular senescence refers to the stable proliferation arrest induced by various stresses. With aging, such cells accumulate in multiple tissues, including joints (Jeon et al., 2017; Xu et al., 2017). We and others have developed a new class of drugs, which can specifically kill senescent cells (senolytics) and show promise in delaying multiple diseases collectively (Kirkland et al., 2017). In this study, we tested the efficacy of dasatinib plus quercetin (D+Q) (Xu et al., 2018) on TMJ degeneration in aging. D+Q can specifically kill a range of senescent cell types by transiently disabling the senescence‐associated anti‐apoptotic pathways (SCAPs) that protect senescent cells from their pro‐apoptotic microenvironment (Zhu et al., 2015). We chose D+Q rather than other senolytic agents in this study because: (1) D+Q seems to eliminate more senescent cell types than other available senolytics (Kirkland & Tchkonia, 2017); (2) D+Q has been extensively examined by us and others and was shown to improve a range of age‐related tissue dysfunction, indicating that D+Q may slow the fundamental aging process; (3) D+Q can extend the lifespan of aged mice, minimizing the risk of long‐term life‐threatening toxic effects at least in mice (Xu et al., 2018); (4) D+Q is the only senolytic agent so far shown to be relatively safe with intermittent oral administration in older human subjects (Justice et al., 2019) and able to kill human senescent cells in vivo (Hickson et al., 2019). Therefore, the clinical translational potential of D+Q in various age‐related diseases could be high. Indeed, a number of clinical trials are currently underway to examine the efficacy of D+Q in human subjects. The benefits of D+Q on various age‐related pathological conditions have been extensively examined by us and others in recent years, including physical dysfunction (Xu et al., 2018), osteoporosis (Farr et al., 2017), insulin resistance(Palmer et al., 2019), Alzheimer's disease (Zhang et al., 2019), and lifespan reduction (Xu et al., 2018). However, the effect of D+Q on TMJ degeneration is still unknown.

To examine whether D+Q could be beneficial to TMJ dysfunction with aging, we treated 23‐month‐old (old) and 4‐month‐old (young) male C57BL/6 mice with D+Q (5 mg/kg+50 mg/kg by oral gavage) or vehicle (V) one course biweekly (Xu et al., 2018), each course containing 3 consecutive days of daily D+Q treatment. One week after 3 courses (over 6 weeks) of drug treatment, we collected TMJs from each mouse. We also treated 4‐month‐old and 24‐month‐old female C57BL/6 mice with D+Q to investigate potential sex differences. Similar to other aged tissues, D+Q significantly reduced the p21 (a key cellular senescence marker), Rela (v‐rel avian reticuloendotheliosis viral oncogene homolog A, a key subunit for NF‐κB pathway) and senescence‐related matrix metalloproteinase 13 (Mmp13) levels (Jeon et al., 2017; Wang et al., 2020) in old TMJs (Figure 1a and Figure S1), demonstrating the efficacy of these senolytics. We first performed histomorphometric analysis (Chen et al., 2020) on these TMJs. The MCC of the TMJ consists of both mineralized cartilage and non‐mineralized cartilage (Bonnevie & Mauck, 2018; Utreja et al., 2016). Cartilage degeneration happens when the mineralized cartilage zone migrates toward the non‐mineralized cartilage, that is, the area of non‐mineralized cartilage is decreased. Using alkaline phosphatase (AP) staining (an enzymatic indicator of mineralization), we found that the distance from the superficial to mineralized layers of cartilage (AP distance) decreased with aging. D+Q did not significantly alter the AP distance in either young or old TMJs (Figure S2A,B), indicating that D+Q did not induce mineralized cartilage encroachment. Importantly, D+Q increased AP signal in the MCC area in old TMJs (for both sexes), similar to the level seen in young TMJs (Figure S2C). These findings suggest that D+Q can preserve mineralized cartilage, which is essential to transmit the masticatory and other functional loads from non‐mineralized cartilage to subchondral bone (Nicholson et al., 2006; Sobue et al., 2011). Moreover, we observed an age‐related decline of AP signal in subchondral bones from V‐treated old TMJs, as well as significantly increased AP activity in TMJs of D+Q treated old mice (both sexes) (Figure 1b,c), indicating increased bone formation. We next assessed the osteoclast activity using TRAP (tartrate‐resistant alkaline phosphatase) staining. Unlike skeleton bone (Farr et al., 2017), TRAP activity was reduced in TMJ with aging (Figure 1e). Similar to AP signal, D+Q partially rescued the age‐related osteoclast reduction in the subchondral bones in old TMJs (Figure 1d,e). Since bone mineral density and bone volume were also increased in these TMJ (Figure 2e‐g), this indicates that the coupled osteoblast activity and resulting bone formation is likely increased. Interestingly, we observed TRAP signal in MCC in D+Q‐treated old TMJs while the TRAP signal was limited to subchondral bone in all V‐treated old TMJs. Increased TRAP expression in MCC is also observed in young TMJs (Chen et al., 2020; Dutra et al., 2018) and may be related to the increased remodeling of the MCC, which is a hallmark of young TMJs. These data suggest that intermittent administration of D+Q can improve bone turnover (both bone formation and resorption) in old TMJs.

**FIGURE 1:**
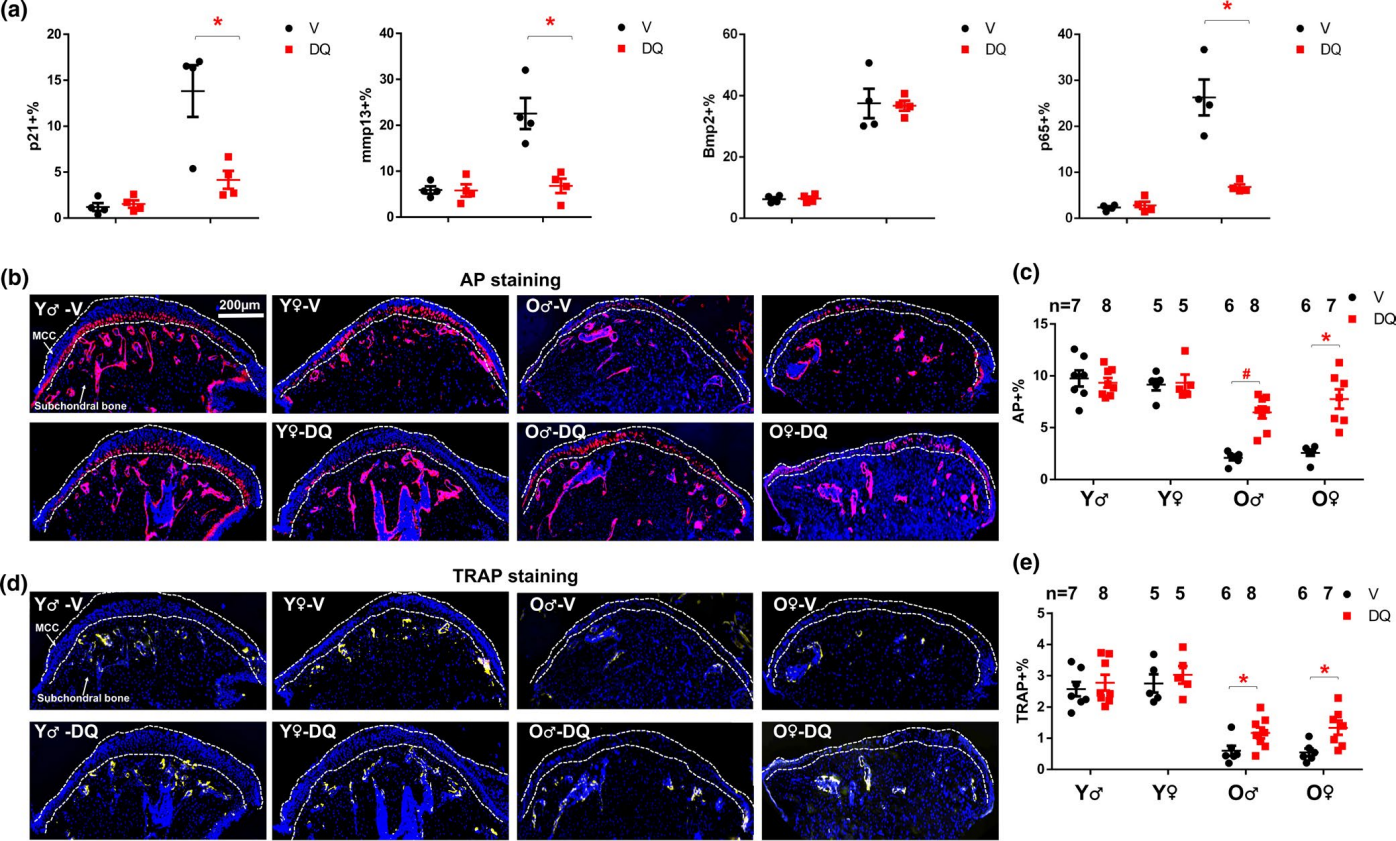
D+Q improved subchondral bone turnover in old TMJ. (a) Percentages of *p21*‐positive cells, Mmp13‐positive cells, Bmp2‐positive cells, and Rela‐positive cells among all cells in MCC region were shown. Representative images of AP staining (b) and TRAP staining (d) were shown. (c) AP‐positive area over total area and (e) TRAP‐positive area over total area was shown. All data were shown as means ±s.e.m. *, *p*<0.05 for two‐way ANOVA; ^#^, *p* < 0.05 for Student's *t* test

**FIGURE 2:**
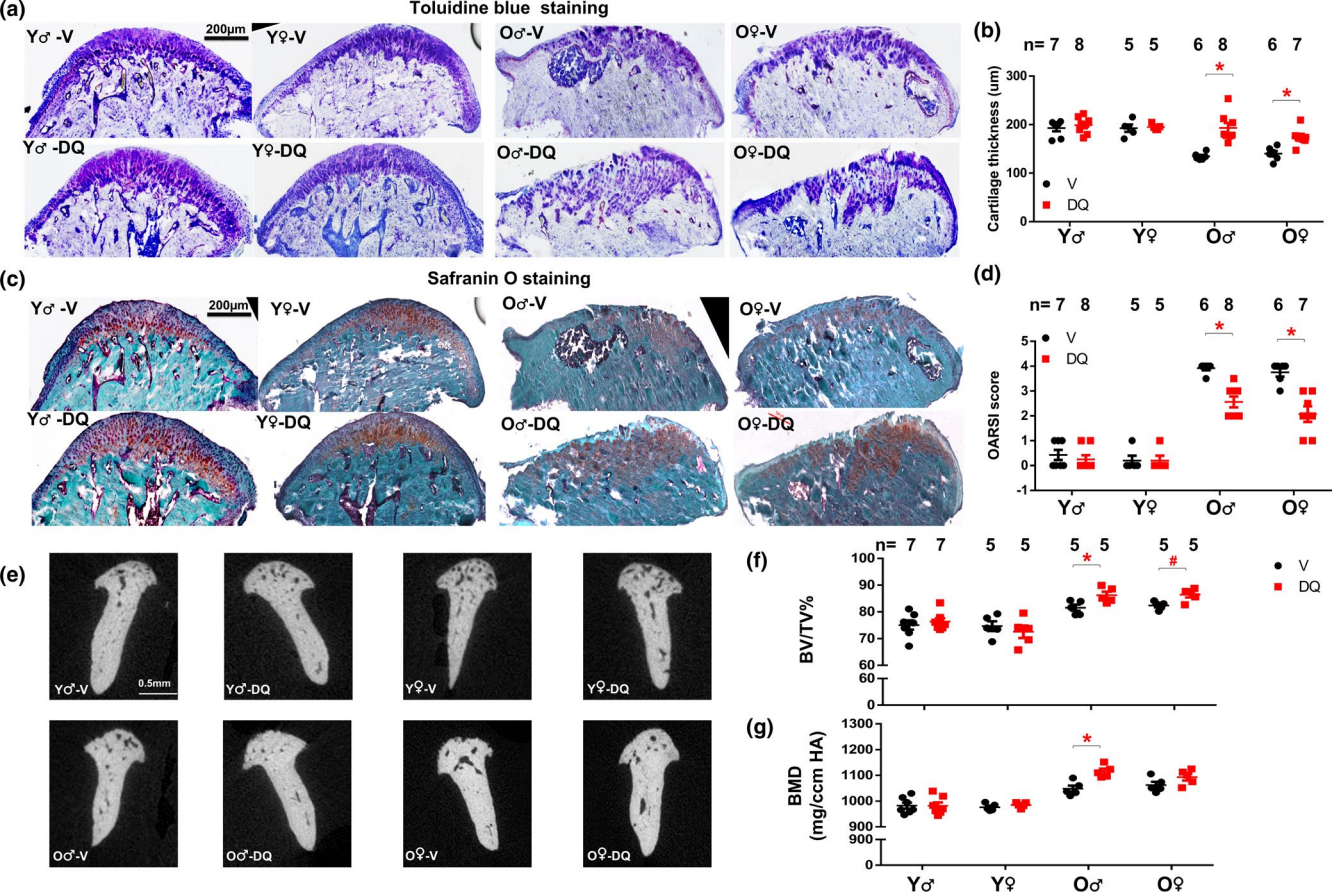
D+Q increased cartilage thickness and decreased OARSI score in old TMJ. Representative images of Toluidine blue staining (a) and Safranin O staining (c) were shown. (b) Cartilage thickness was shown. (d) OARSI score was assessed and shown. (e) Representative images of µCT. (f) Bone volume over total volume and (g) Bone density was shown. All data were shown as means ±SEM. *, *p* < 0.05 for two‐way ANOVA; ^#^, *p* < 0.05 for Student's *t* test

We next performed Safranin O and toluidine blue (TB) staining to assess the cartilage thickness and overall pathological states. Safranin‐O stains the cartilage orange to red and nuclei will be stained black. The background (usually bone) will be stained green. TB stains the cartilage matrix/proteoglycans in the matrix intense blue to light blue. D+Q‐treated old TMJs contained more proteoglycan content in MCC and had increased cartilage thickness when compared to V‐treated old mice. D+Q had no detectable effects on young TMJs (Figure 2a‐c). Notably, we observed that D+Q increased cartilage thickness by more than 30% in old TMJs within 5–8 weeks (Figure 2b). Since TMJ cartilage thickness declines gradually with aging (Chen et al., 2020), our data support less degeneration with DQ treatment and plausible reasons could be regeneration or prevention of degeneration of the TMJ cartilage. This could be clinically significant, since the current barrier to success in TMJ degeneration treatment is that TMJs fail to regenerate the diseased cartilage tissue that could recapitulate the cellular composition, structure, and load‐bearing capacity of healthy joint cartilage. Using the OARSI cartilage histopathology assessment system (Pritzker et al., 2006), we observed minor to no pathological conditions in both V and D+Q treated young TMJs (using Safranin O staining) while D+Q significantly reduced OARSI scores in old TMJs (Figure 2d), indicating less severe TMJ abnormality and better cartilage integrity.

Lastly, we used micro‐computed tomography (µCT) to assess quantitative changes in the mandibular condyle of these mice. Here, we found that both bone volume and bone mineral density (BMD) increased with aging. Consistent with improved bone turnover, higher bone volume was observed in D+Q‐treated old TMJs for both sexes; however, higher BMD was only observed in D+Q‐treated old male but not female TMJs (Figure 2e‐g). 3D surface topography of the mandibular condyle showed more porous or trabecular bone in the young TMJs, which was not affected by D+Q. In old TMJs, we observed craters in the vehicle group (Figure S2D, indicated by the arrows), but not in the D+Q group, consistent with our bone volume and BMD measurements (Figure 2e‐g). It is possible that D+Q might increase BMD in MCC in old TMJs, which might indicate cartilage calcification. Since D+Q did not alter AP distance (Figure S2B), it excludes the possibility that non‐mineralized cartilage is encroached by mineralized cartilage in these D+Q treated TMJs. In summary, these findings suggest that D+Q can increase bone volume and density in old TMJ, similar to its effect on skeletal bones in old mice (Farr et al., 2017).

In summary, our findings demonstrate that biweekly administration of D+Q improves subchondral bone turnover, alleviates cartilage degeneration and pathological conditions, and increases bone volume in TMJs from old mice. Of note, the benefits on the TMJ cartilage by senescent cell clearance is similar to the knee cartilage, as previous studies showed that transplantation of senescent cells impaired knee cartilage morphology (Xu et al., 2017) and clearance of senescent cells in aged or osteoarthritis knee alleviated cartilage pathology (Jeon et al., 2017). Despite slightly different starting ages (23 vs 24 months), we observed similar changes in TMJs by D+Q in both male and female mice, indicating little sex difference as regards the benefits of D+Q in TMJs in aged animals. It is possible that D+Q alleviates TMJ dysfunction through mechanisms beyond the clearance of senescent cells, since both D and Q have pleiotropic effects. In this study, D+Q treatment was intermittent with 3 doses every 2 weeks and TMJs were collected 7 days after last dose of D+Q. Because both D and Q have short elimination half‐lives (<12 hours) (Xu et al., 2018), the effect we observed was most likely due to mechanisms that are long‐lasting and do not require continuous presence of the drugs to inhibit or activate certain enzymes or pathways. Moreover, D+Q seems to only have effects on old mice, but not on young mice (which contain few senescent cells), indicating age‐specific benefits. All these pieces of evidence are consistent with the possibility that D+Q mitigates age‐related TMJ degeneration at least partially through eliminating senescent cells. It is still unclear how D+Q increases cartilage thickness and bone turnover (increased AP and TRAP signals) in old TMJs. We found that Bmp2 level increased with aging (consistent with previous findings (Chen et al., 2020)), while DQ has little effect on Bmp2 level in either young or old TMJs (Figure 1a), indicating that D+Q might not work through Bmp2 pathway. Future experiments are needed to further investigate these underlying mechanisms. Although the TMJ morphological features differ between human and mice, our study here provides preclinical evidence that D+Q has potential to improve TMJ tissue homeostasis in mice with aging and likely lays the foundation for clinical trials to treat TMJ degeneration in old age.

## CONFLICTS OF INTEREST

M.X., S.Y., Y.Z., and P.C. have financial interests related to this research. US Provisional Patent on D+Q on old TMJ (63/142,318) has been filed by UConn Health on 1/27/2021. Patents on senolytic drugs (including PCT/US2016/041646, filed at the US Patent Office) are held by Mayo Clinic.

## AUTHOR CONTRIBUTIONS

Y.Z., S.Y., and M.X. conceived and designed the study. I.M.A. performed most D+Q administration. Y.Z., P.C., K.W., and S.M. performed all the experiments. N.S.G. and G.A.K. contributed to the data analysis and the manuscript preparation. Y.Z. and M.X., wrote the manuscript with input from all coauthors. M.X. and S.Y. oversaw all experimental design, data analysis, and manuscript preparation.

## Supporting information

Supplementary Material

Figure S1

Figure S2

## Data Availability

The data are available from the corresponding author upon reasonable request.
