# Supplementary material for: Senolytics alleviate the degenerative disorders of temporomandibular joint in old age
Source: Aging Cell. 2021 Jun 8;20(7):e13394. doi: 10.1111/acel.13394 (PMC8282237; doi:10.1111/acel.13394)
Supplement: Supplementary file 1 — Supplementary Material [file ACEL-20-e13394-s002.pdf]

Supporting information

Materials and Methods

Mouse models and drug treatments. All animal experiments were performed according to protocols approved by the Institutional Animal Care and Use Committee (IACUC) at UConn Health. Wild-type C57BL/6 mice (both male and female) were obtained from the National Institute on Aging (NIA) and maintained in a pathogen-free facility at 23–24 °C under a 12-h light, 12-h dark regimen with free access to a standard mouse diet (Teklad global 18% protein, Envigo #2918, Indianapolis, IN) and water. Dasatinib and quercetin were dissolved in vehicle containing 60% Phosal 50PG, 30% PEG-400, and 10% Ethanol. Mice were administrated either vehicle or D+Q (Dasatinib: 5mg/kg body weight + Quercetin: 50mg/kg) via oral gavage for 3 consecutive days, every two weeks for 6 weeks (total 9 doses). This treatment regimen has been shown by us and others to be effective for elimination of senescent cells *in vivo*, and improvement of various age-related conditions(Farr et al., 2017; Xu et al., 2018). Dasatinib was purchased from LC Laboratories (Woburn, MA). Quercetin was purchased from Sigma-Aldrich (St Louis, MO).

Tissue Preparation and Histological Sectioning. The MCC along with the subchondral bone was dissected free by cutting the muscular attachment without scrapping the cartilage and was fixed for 24 hours in 10% formalin, followed by incubation in 30% sucrose in PBS overnight. Specimens were embedded in cryo-medium (Thermo Shandon, Pittsburgh, PA, USA) using disposable base molds (Thermo Shandon) and were stored at -80°C before sectioning. Frozen sagittal sections of the condyles (6μm) were prepared using a Leica cryostat (Nussloch, Germany) and were then transferred to slides.

Micro-CT. The MCC and subchondral bone were analyzed using micro-computerized tomography (micro-CT) (SCANCO Medical AG, Bruttisellen, Switzerland) as previously published(Chen et al., 2020). The samples were scanned in 70% alcohol and 55 kV and 145 μA was used to acquire serial tomographic projections, with a voxel size of 6μm and 1000 projections per rotation were collected at 300,000 μs. An automated algorithm using local threshold segmented the reconstructed grey scale images to distinguish calcified tissue from non-calcified tissue. The mushroom shaped head of the condyle was our region of interest. Bone volume over total volume (BV/TV%) and bone tissue density (mg/ccm HA) were assessed.

Histological staining and quantification. Tissue sections were stained for Tartrate Resistant Acid Phosphatase (TRAP) using ELF97 (Life Tech, Waltham, MA, USA), which generates a yellow fluorescent signal. After imaging for TRAP, the same slide was stained for alkaline phosphatase activity using a fluorescent fast red substrate (Sigma) and for cell nuclei using DAPI (Thermo Fisher Scientific, Waltham, MA, USA) then reimaged. After that, the slide was rinsed in distilled water and stained with TB (IHC WORLD, LLC; Woodstock, MD, USA)) to examine proteoglycans, and reimaged using bright field microscopy. TB stains the cartilage matrix/proteoglycans in the matrix intense blue to light blue. Finally sections were stained for safranin O (IHC WORLD, LLC; Woodstock, MD, USA). The cartilage will stain orange to red and nuclei will be stained black. The background (usually bone) will be stained green. We examined TRAP and AP activity in the MCC and subchondral bone by counting the number of yellow and red pixels dividing by the total number of pixels in the whole region. AP distance was measured by assessing the distance from superficial layer of the cartilage to

mineralized cartilage layer (AP positive layer) in 10 different locations throughout the MCC. Cartilage thickness was measured in TB-stained sections. The distance from the outer cellular layer of the MCC to the tidemark was measured and normalized to the scale bar in 10 different locations throughout the entire MCC, and averaged absolute thickness (µm) was shown. Immunostaining for MMP13 (#39012, targeting both latent and active form, Abcam, Cambridge, UK), Rela (#231481, Abcam), Bmp2 (#14933, Abcam) and p21 (sc-6246, Santa Cruz Biotechnology, Dallas, TX) were performed as previously published (Chen et al., 2020; Wang et al., 2020). For all staining images, 3-4 images were taken per mouse (covering most of the MCC region), and total of 300-800 cells per mouse were counted for quantification.

OARSI score assessment. OARSI score was assessed according to the OARSI cartilage histopathology assessment system (Pritzker et al., 2006) in safranin O-stained sections. Single examiner did the measurement of the OARSI scores. The examiner was blinded to the groups and did the measurement twice (3 days apart) on the randomly generated samples provided to him. Cohen's kappa statistic for the intra-examiner reliability was 0.95.

Statistical analysis. Both Two-Way ANOVA with Fisher's LSD test and unpaired Student's t-test were used to compare 2 groups.

Author Contributions

Y.Z., S.Y., and M.X. conceived and designed the study. I.M.A. performed all D+Q administration. Y.Z., P.C., K.W., S.M. performed all the experiments. N.S.G., and G.A.K. contributed to the data analysis and the manuscript preparation. Y.Z., and M.X., wrote the manuscript with input from all coauthors. M.X. and S.Y. oversaw all experimental design,

data analysis, and manuscript preparation.

Supplementary References

Chen, P.J., Dutra, E.H., Mehta, S., O'Brien, M.H., and Yadav, S. (2020). Age-related changes in the cartilage of the temporomandibular joint. Geroscience *42*, 995-1004.

Farr, J.N., Xu, M., Weivoda, M.M., Monroe, D.G., Fraser, D.G., Onken, J.L., Negley, B.A., Sfeir, J.G., Ogrodnik, M.B., Hachfeld, C.M.*, et al.* (2017). Targeting cellular senescence prevents age-related bone loss in mice. Nature medicine *23*, 1072-1079.

Pritzker, K.P., Gay, S., Jimenez, S.A., Ostergaard, K., Pelletier, J.P., Revell, P.A., Salter, D., and van den Berg, W.B. (2006). Osteoarthritis cartilage histopathology: grading and staging. Osteoarthritis Cartilage *14*, 13-29.

Wang, B., Liu, Z., Chen, V.P., Wang, L., Inman, C.L., Zhou, Y., Guo, C., Tchkonia, T., Rowe, D.W., Kuchel, G.A.*, et al.* (2020). Transplanting cells from old but not young donors causes physical dysfunction in older recipients. Aging Cell, e13106.

Xu, M., Pirtskhalava, T., Farr, J.N., Weigand, B.M., Palmer, A.K., Weivoda, M.M., Inman, C.L., Ogrodnik, M.B., Hachfeld, C.M., Fraser, D.G.*, et al.* (2018). Senolytics improve physical function and increase lifespan in old age. Nature medicine *24*, 1246-1256.

Fig.S1 D+Q reduced senescence-associated markers in old TMJs. Representative images of p21 staining (Red: p21; Blue: DAPI), Mmp13 (Green: Mmp13; Blue: DAPI), Bmp2 (Red: Bmp2; Blue: DAPI), and Rela (Red: Rela; Blue: DAPI).

Fig.S2 D+Q improved mineralization in old TMJ. (A) Schematic for AP distance measurement. (**B**) AP distance and (**C**) AP positive area in MCC was shown. All data was shown as means ± s.e.m. *, $p<0.05$ for Two-Way ANOVA. (**D**) Representative images of 3D surface topography of the mandibular condyle. Arrows indicate craters.
